# Human CD4^+^ T Cell Responses to the Dog Major Allergen Can f 1 and Its Human Homologue Tear Lipocalin Resemble Each Other

**DOI:** 10.1371/journal.pone.0098461

**Published:** 2014-05-29

**Authors:** Aino L. K. Liukko, Tuure T. Kinnunen, Marja A. Rytkönen-Nissinen, Anssi H. T. Kailaanmäki, Jukka T. Randell, Bernard Maillère, Tuomas I. Virtanen

**Affiliations:** 1 Department of Clinical Microbiology, Institute of Clinical Medicine and Biocenter Kuopio, University of Eastern Finland, Kuopio, Finland; 2 Institute of Dentistry, School of Medicine, University of Eastern Finland, Kuopio, Finland; 3 Department of Pulmonary Diseases, Kuopio University Hospital, Kuopio, Finland; 4 Commissariat à l'Energie Atomique, Institut de Biologie et de Technologies, Service d'Ingénierie Moléculaire des Protéines, Gif Sur Yvette, France; Albert Einstein Institute for Research and Education, Brazil

## Abstract

Lipocalin allergens form a notable group of proteins, as they contain most of the significant respiratory allergens from mammals. The basis for the allergenic capacity of allergens in the lipocalin family, that is, the development of T-helper type 2 immunity against them, is still unresolved. As immunogenicity has been proposed to be a decisive feature of allergens, the purpose of this work was to examine human CD4^+^ T cell responses to the major dog allergen Can f 1 and to compare them with those to its human homologue, tear lipocalin (TL). For this, specific T cell lines were induced *in vitro* from the peripheral blood mononuclear cells of Can f 1-allergic and healthy dog dust-exposed subjects with peptides containing the immunodominant T cell epitopes of Can f 1 and the corresponding TL peptides. We found that the frequency of Can f 1 and TL-specific T cells in both subject groups was low and close to each other, the difference being about two-fold. Importantly, we found that the proliferative responses of both Can f 1 and TL-specific T cell lines from allergic subjects were stronger than those from healthy subjects, but that the strength of the responses within the subject groups did not differ between these two antigens. Moreover, the phenotype of the Can f 1 and TL-specific T cell lines, determined by cytokine production and expression of cell surface markers, resembled each other. The HLA system appeared to have a minimal role in explaining the allergenicity of Can f 1, as the allergic and healthy subjects' HLA background did not differ, and HLA binding was very similar between Can f 1 and TL peptides. Along with existing data on lipocalin allergens, we conclude that strong antigenicity is not decisive for the allergenicity of Can f 1.

## Introduction

Type I allergic immune response is known to be mediated by CD4^+^ T-helper type 2 (Th2) lymphocytes that, through the production of cytokines, such as interleukin (IL)-4, IL-5 and IL-13, orchestrate allergen-specific IgE synthesis and eventually eosinophilic inflammation in the target organs [1]. Although it is well accepted that interactions of several environmental and genetic factors are needed to induce such a development [1], it is still largely unclear what role an allergenic protein plays in the process. It is reasonable to hypothesize, though, that allergens possess specific allergenic properties because allergic sensitization is mostly manifested by the production of IgE specific to only a few selected proteins present in the allergen source [2]–[4]. Nevertheless, exposure to low to moderate concentrations of these proteins through airways leads to allergic sensitization and overt respiratory allergy in susceptible individuals [4].

In the human environment, especially indoors, the allergens of the lipocalin family are of great importance, as they contain most of the significant respiratory allergens from dog, cat, mouse, rat, guinea pig, rabbit, horse and cow [5]–[7]. Many of the mammalian lipocalin allergens are classified as major allergens. These include, for example, dog allergen Can f 1 [8], horse allergen Equ c 1 [9], and cow allergen Bos d 2 [10]. In addition to mammalian allergens, four arthropodan allergens [11]–[14] as well as a food allergen Bos d 5 (β -lactoglobulin) [15] are lipocalins [6]. The lipocalin family also contains human endogenous proteins [16], such as the homologue of Can f 1, tear lipocalin (lipocalin-1/von Ebner's gland protein), which exhibits an amino acid identity of about 60% with Can f 1 [6]. Several other lipocalin allergens are also known to exhibit homologies of 30–60% with human endogenous lipocalins [5], [17].

One necessary requirement for the development of allergen-specific Th2-deviated immune response and subsequent IgE production is the recognition of the allergenic protein by the adaptive immune system [1]. In this process, CD4^+^ T-helper cells recognize allergen peptides on antigen-presenting cells (APCs) through their antigen receptors, T cell receptors (TCRs). Therefore, any allergen, as antigens in general, needs to be different from the immunological self, i.e. antigenic/immunogenic, so that a productive immune response can take place [18]. In line with this immunological paradigm, it has recently been proposed that immunogenicity is a major factor accounting for the allergenicity of proteins [19].

Although this view is tempting, it is not straightforward to reconcile it with the available data on allergens. For example, it is obvious that human environment is full of proteins of different immunogenic potential but only a minor fraction of them are allergenic. This fact is illustrated by an analysis in the Pfam database which demonstrated that allergens were found in only 2% of more than 9300 protein families and that 10 of these families contained more than 40% of the allergens analyzed [20]. Moreover, some sources, especially bacteria, which are phylogenetically distant from humans contain few major allergens [19]. Allergens, on the other hand, are rarely homologous to bacterial proteins [19], [21]. There are also data indicating that even within the same allergen source, such as mites [22], cockroaches [23] or pollen [24], [25], the antigenicity of proteins, in terms of strength of T cell response *in vitro*, is not linked to allergenicity. Therefore, it seems that even if antigenicity/immunogenicity is a prerequisite for the adaptive immunity to arise, its connection to the allergenicity of proteins in general is less clear.

The purpose of this study was to compare the antigenic properties of the major dog allergen Can f 1 and its human homologue, tear lipocalin (TL), by analyzing human CD4^+^ T cell responses against them *in vitro*. We observed that the quantitative and qualitative characteristics of Can f 1 and TL peptide-specific T cell lines generated from both dog-allergic and dog dust-exposed healthy subjects resembled each other in a surprising manner, suggesting that the antigenicity of Can f 1 is weak and probably does not explain the allergenicity of the protein.

## Methods

### Ethics Statement

The study was approved by the Ethics Committee of the Kuopio University Hospital with the permit number 182/1999, valid until the end of 2015. Written informed consent was obtained from all the participants in the study.

### Subjects

Peripheral blood samples were collected from 14 clinically dog-allergic patients and from 15 healthy non-atopic dog-dust exposed individuals (Table S1) for this study. The allergic subjects were characterized at the Pulmonary Clinic of Kuopio University Hospital, as described in detail previously [26], [27]. In brief, they exhibited a positive dog UniCAP result (FEIA; Pharmacia, Uppsala, Sweden; >0.7 kU/L), positive SPTs (≥3 mm) with a commercial dog epithelial extract (ALK Abellò, Hørsholm, Denmark) and recombinant Can f 1 at 2.5 µg/ml [26], [27], and a positive clinical history for asthma and other allergic symptoms with exacerbation of the symptoms under dog dust exposure (Table S1). The nonallergic subjects reported no medical history of atopy or allergic symptoms of any kind. HLA class II genotyping for the DQ and DR alleles of the subjects was performed in the Clinical Laboratory of Finnish Red Cross Blood Service (Helsinki, Finland [28]) or in the Immunogenetics Laboratory of University of Turku (Turku, Finland [29]) with PCR-SSO and PCR-SSP methods (Table S1). The distribution of the HLA class II alleles did not differ between allergic and healthy subjects (Fisher's exact test, p>0.05).

### Antigens

Nine Can f 1 (pC1–pC9) and nine homologous tear lipocalin (pTL1–pTL9) peptides along the aligned protein sequences were selected (Table 1) based on the verified T cell epitopes of Can f 1 [30] and on the ProPred predictions of HLA-DR-binding sequences (http://www.imtech.res.in/raghava/propred/ [31]) in Can f 1 and TL (Fig. S1). The peptides, ranging from 16 to 19 amino acids (aa), were synthesized at ≥80% purity by GL Biochem Ltd., China. For the induction of T cell lines (TCLs), the peptides were arranged in pools, each of the six pools containing three Can f 1 or TL peptides as follows: pC1+pC4+pC7, pC2+pC5+pC8, pC3+pC6+pC9, pTL1+pTL4+pTL7, pTL2+pTL5+pTL8, and pTL3+pTL6+pTL9. The influenza hemagglutinin (HA) peptide (amino acids 306–318) [32] was also produced by GL Biochem Ltd.

**Table 1:** Peptides used in the study.

Description	Abbr.	Sequence	Length (aa)
Can f 1			
p15–30	pC1	GKWYLKAMTADQEVPE	16
p31–48	pC2	KPDSVTPMILKAQKGGNL	18
p48–64	pC3	LEAKITMLTNGQCQNIT	17
p73–88	pC4	PGKYTAYEGQRVVFIQ	16
p81–96	pC5	GQRVVFIQPSPVRDHY	16
p95–110	pC6	HYILYCEGELHGRQIR	16
p107–123	pC7	RQIRMAKLLGRDPEQSQ	17
p125–140	pC8	ALEDFREFSRAKGLNQ	16
p141–156	pC9	EILELAQSETCSPGGQ	16
Tear lipocalin			
p15–30	pTL1	GTWYLKAMTVDREFPE	16
p31–49	pTL2	MNLESVTPMTLTTLEGGNL	19
p49–65	pTL3	LEAKVTMLISGRCQEVK	17
p74–89	pTL4	PGKYTADGGKHVAYII	16
p82–97	pTL5	GKHVAYIIRSHVKDHY	16
p96–111	pTL6	HYIFYCEGELHGKPVR	16
p108–124	pTL7	KPVRGVKLVGRDPKNNL	17
p126–141	pTL8	ALEDFEKAAGARGLST	16
p142–158	pTL9	ESILIPRQSETCSPGSD	17
Influenza HA			
p306–318	HA	PKYVKQNTLKLAT	13

### Generation of peptide-specific CD4^+^ T cell lines with the split-well method

After isolating peripheral-blood mononuclear cells (PBMCs) by Ficoll-Paque Plus density gradient centrifugation (GE Healthcare Biosciences, Uppsala, Sweden), CD4^+^CD25high regulatory T cells were depleted with anti-CD25-magnetic beads (Miltenyi Biotec, Bergisch Gladbach, Germany), to enhance the induction of both autoreactive and allergen-reactive CD4^+^ T cell responses, as previously described [33], [34].The CD25-depleted PBMCs were seeded in 96-well U-bottomed plates (Corning Incorporated, Corning, USA; 10^5^ PBMCs per well) in the presence of pooled Can f 1 or TL peptides (each peptide at 2 µM, 30 wells per a peptide pool), or the HA peptide (30 wells) in a 150 µl volume of RPMI 1640 culture medium supplemented with 2 mM L-glutamine, 20 µM 2-mercaptoethanol, 1 mM sodium pyruvate, nonessential amino acids, 100 IU/ml penicillin, 100 µg/ml streptomycin, 10 mM HEPES (all from Lonza, Verviers, Belgium) and 5% inactivated human AB serum (Sigma-Aldrich, St. Louis, MO). On day 5, 50 µl of fresh medium supplemented with 10 IU/ml of IL-2 (Strathmann Biotech, Hannover, Germany) was added to the cultures. On day 7, half of the culture medium was replaced without IL-2. On day 10, half of the cells in the cultures were split into daughter plates for testing specificity by stimulating the cells with or without the pooled peptides (each peptide at 2 µM) together with autologous γ-irradiated (3000 rad) PBMCs (5×10^5^) as antigen-presenting cells. After an incubation of 72 h, [3H]thymidine was added (1 µCi per well; GE Healthcare, Little Chalfont, UK), and after an additional 16 h, the cells were harvested onto glass fiber filters on day 14 (Wallac, Turku, Finland). Radioactivity was measured by scintillation counting (Wallac Micro Beta 1540) and the results were expressed as counts per minute (CPM). The stimulation indices (SI; CPM in the presence of antigen divided by CPM in the absence of antigen) and CPM differences between stimulated and unstimulated wells (ΔCPM) were determined. On the same day, positive cultures (ΔCPM>2000 and SI>2) were restimulated in 48-well plates with autologous γ-irradiated PBMCs (10^6^/well), 2 µM of the peptides and 25 IU/ml of IL-2. On day 28, the established TCLs were propagated once more with peptides, IL-2 and PBMCs. After stimulations, half of the culture medium was replaced with fresh medium supplemented with IL-2 every 2–3 days. The peptide specificity of TCLs was defined as described below.

### Proliferation assays

T cell proliferation assays were set in duplicates in 96-well U-bottomed plates with 2.5×10^4^ cultured T cells, 5×10^4^ autologous γ-irradiated (3000 rad) PBMCs as APCs, and antigens as indicated. In order to determine the epitope specificity of the established TCLs induced with peptide pools, the T cell lines were tested with each of the three individual peptides in the pool at 10 µM on day 28. Proliferation was measured by radionuclide uptake (see above). The TCLs that showed a positive response (ΔCPM>1000, SI>2) upon stimulation with a peptide were regarded as peptide-specific TCLs and were further analyzed in the study. If reactivity to two different peptides was observed in the cultures induced with a peptide pool, the TCL was considered specific to each of these peptides in the analyses. In all, only 10 and 7 peptide pool-induced cultures from allergic and nonallergic subjects, respectively, showed reactivity to two different peptides. No culture showed reactivity to all three peptides in the pool. On day 42, peptide-specific TCLs were tested with individual Can f 1 peptides, as well as their TL homologues, at several concentrations up to 10 µM. The functional T cell receptor (TCR) avidities of the TCLs for a peptide were assessed by determining effective concentration 50 (EC_50_) values (i.e., the concentration of a peptide needed to induce a half-maximal proliferative response) from the dose-response curves of individual TCLs.

### Phenotypic analyses of the generated CD4^+^ T cell lines

Upon defining the specificity of the established TCLs to individual peptides (see above), supernatants (100 µl per well) were collected from the proliferation assay plates after a 72 h stimulation with 10 µM of individual peptides on day 31. The supernatants were stored at −70°C until analyzed. The production of IL-4, IL-5, IFN-γ and IL-10 was measured using commercial ELISA kits (DuoSet; R&D Systems; Minneapolis, MN). The expression of CD4 together with CCR4 and CXCR3 was analyzed with a BD FACSCanto II flow cytometer after staining the T cells with specific fluorochrome-labeled monoclonal antibodies (all from BD Biosciences) or corresponding isotype control mAbs on day 42.

### HLA class II peptide-binding assays

A panel of common HLA-DR and HLA-DP4 molecules were immunopurified from homologous EBV cell lines by affinity chromatography using the monomorphic mAbs L243 and B7/21, respectively [35]–[37]. The binding to HLA-DR and HLA-DP4 molecules was assessed by competitive ELISA, as previously described [35]–[37]. To assess the validity of independent experiments, unlabeled forms of the biotinylated peptides were used as reference peptides [35]–[37]. The results were expressed as IC_50_ ratios (ratio between the IC_50_ value of the tested peptide and that of the reference peptide) to take into account the disparity of the binding sensitivity between different HLA molecules.

### Statistical analyses

**Figure 1:**
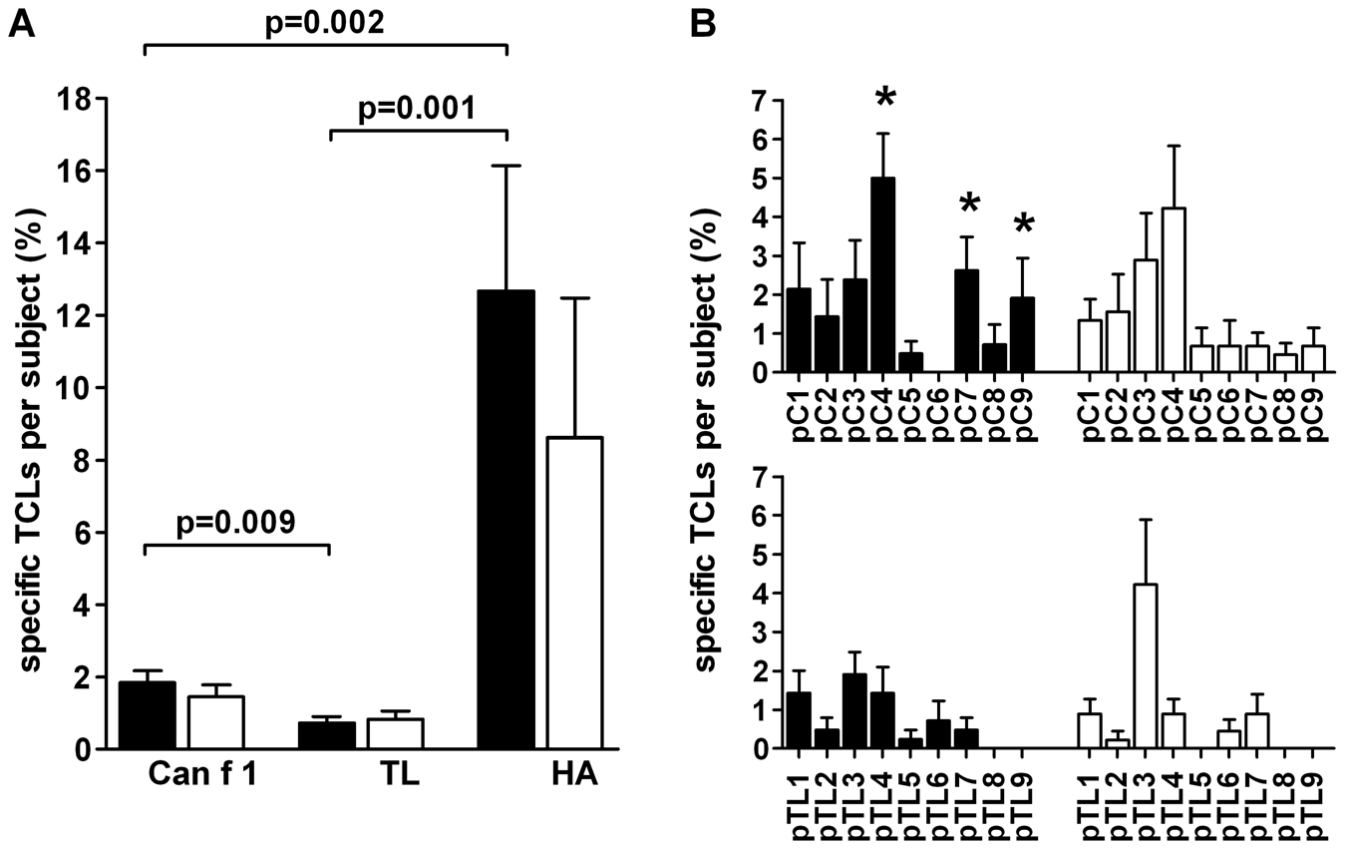
Percentages of positive TCLs induced with Can f 1, TL and HA peptides. (**A**) Percentages of specific TCLs (mean±SEM) obtained per subject with 9 Can f 1 peptides, the corresponding 9 TL peptides, and one HA peptide (out of 30 replicate wells seeded). The TCLs were generated from PBMCs of allergic subjects (▪) and nonallergic subjects (□) with the split well method, as indicated in Methods. (**B**) Percentages of TCLs obtained per subject specific to individual Can f 1 (pC1–pC9) and TL (pTL1–pTL9) peptides. Asterisk (*) indicates Can f 1 peptides that were recognized significantly more frequently than their counterpart TL peptides (p<0.05 for all; See Results).

**Figure 2:**
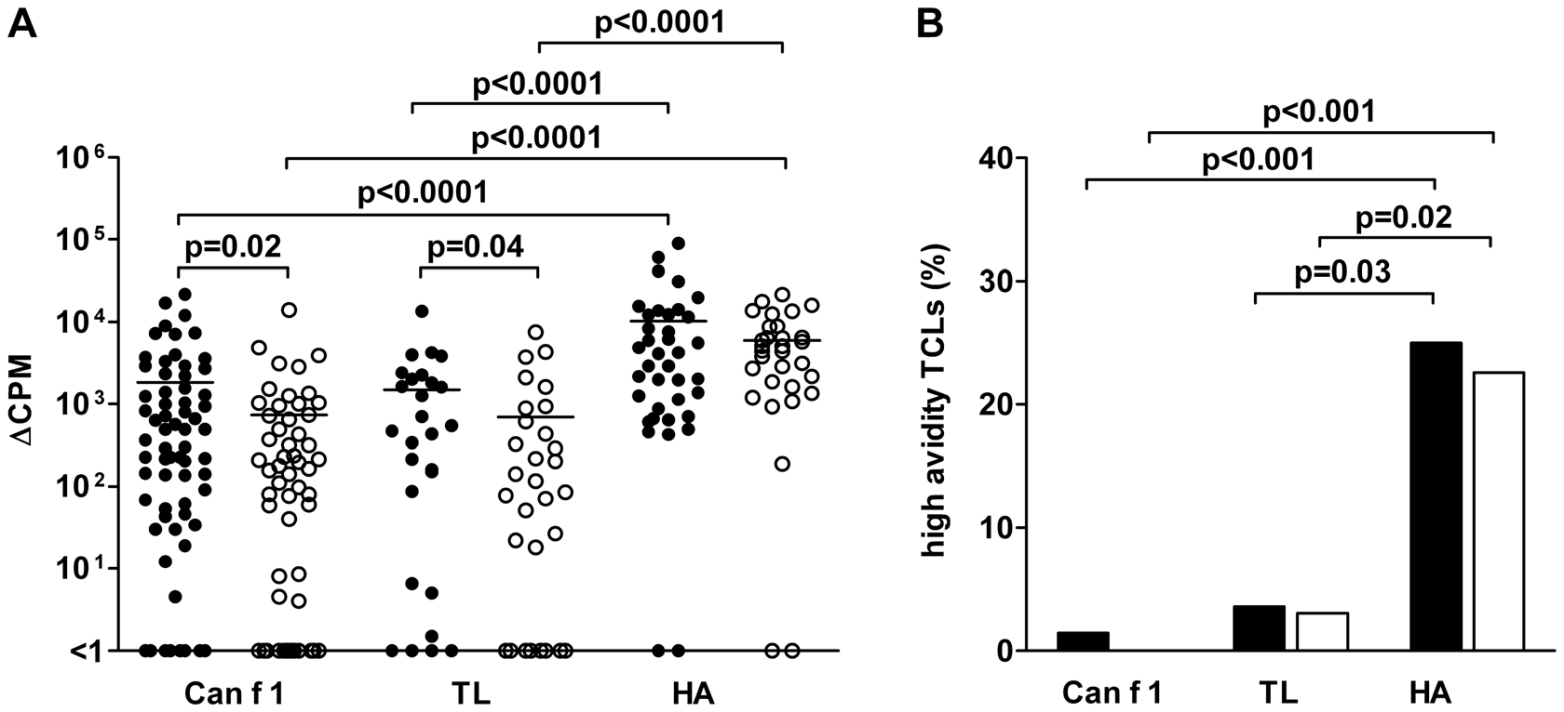
Proliferation and functional avidity of TCLs specific to Can f 1, TL and HA peptides. (**A**) Proliferative responses of TCLs specific to 9 Can f 1 and 9 TL peptides and the HA peptide from allergic (•) and nonallergic subjects (○) upon stimulation with the peptides at 0.1 µM. The responses are expressed as ΔCPM (mean CPM of wells stimulated with the peptide - mean CPM of unstimulated wells). (**B**) Percentages of high-avidity (EC50<0.1 µM) Can f 1, TL and HA-specific TCLs from allergic (▪) and nonallergic (□) subjects.

**Figure 3:**
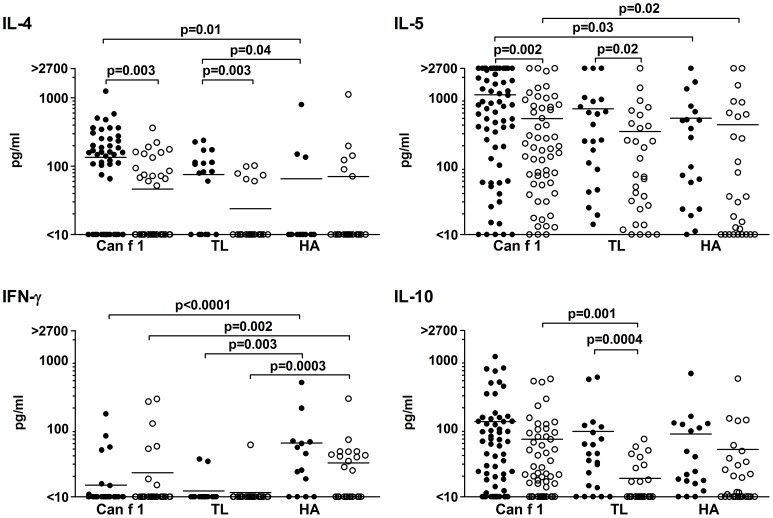
Cytokine production by peptide-specific TCLs. Production of IL-4, IL-5, IFN-γ and IL-10 by the Can f 1, TL and HA-specific TCLs of allergic (•) and nonallergic (○) subjects upon stimulation with the peptides at 10 µM.

**Figure 4:**
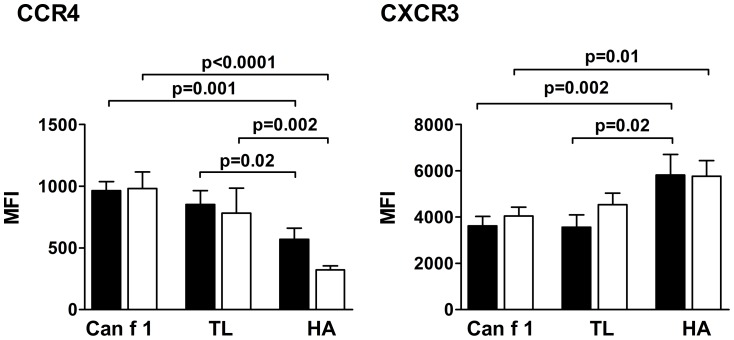
Expression of cell surface markers by peptide-specific TCLs. Expression of the cell surface markers CCR4 and CXCR3 on TCLs specific to Can f 1, TL and HA peptides from allergic (▪) and nonallergic (□) subjects. Results are expressed as mean fluorescence index (MFI)±SEM.

**Figure 5:**
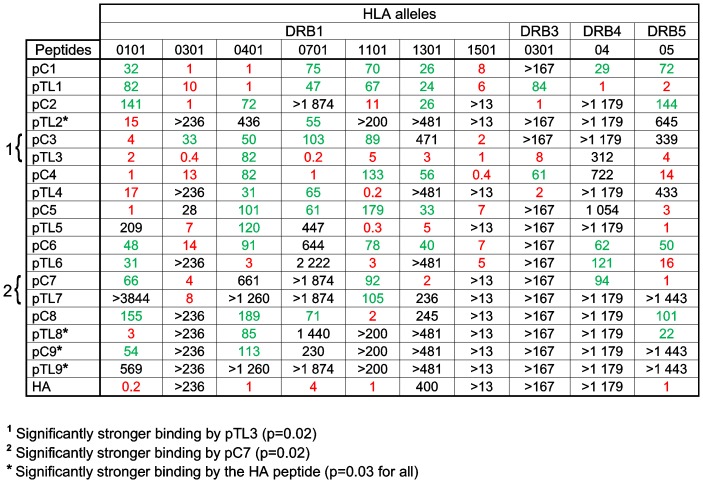
Binding capacities of the peptides pC1-pC9, pTL1-pTL9 and the HA peptide to HLA class II molecules. The binding capacities are expressed as relative binding ratios (IC_50_ of the test peptide divided by IC_50_ of the reference peptide) to take into account the differences in the sensitivity of the binding assays. The reference peptides exhibited the following IC_50_ values: DRB1*0101, 3 nM; DRB1*0301, 423 nM; DRB1*0401, 8 nM; DRB1*0701, 5 nM; DRB1*1101, 50 nM; DRB1*1301, 208 nM; DRB1*1501, 775 nM; DRB3, 60 nM; DRB4, 8 nM; DRB5, 7 nM. Values in red (IC_50_ ratio ≤20) are considered to indicate high affinity binding and values in green (IC_50_ ratio 20–200) moderate affinity binding.

Statistical analyses were conducted using the commercial GraphPad Prism software (Graphpad Software, San Diego, CA). The Mann-Whitney *U* test was used for group analyses of the TCL frequencies (Fig. 1), proliferation responses (Figs. 2A, S3, S4), cytokine responses (Figs. 3, S5) and surface marker expression (Fig. 4). The Fisher's exact test was used for the analysis of the categorical TCL avidity data (Fig. 2B) and the Wilcoxon signed rank test for the analysis of HLA-peptide binding between the subject groups or antigens (Fig. 5). In other cases, the statistical tests were used as indicated. *p* values of 0.05 or less were considered statistically significant.

## Results

### Can f 1 peptides are recognized more frequently than their endogenous TL homologues but less frequently than the viral control peptide

The stimulatory capacity of the dog major allergen Can f 1 and its human homologue TL was first assessed by determining the frequency of peptide-recognizing T cells by generating peptide-specific T cell lines (TCLs) from the PBMCs of allergic and nonallergic subjects. Altogether, we obtained and analyzed 129 TCLs (from 14 allergic and 15 nonallergic subjects) specific to any one of the Can f 1 peptides, 62 TCLs specific to any one of the TL peptides and 69 TCLs (from 10 allergic and 13 nonallergic subjects) specific to the influenza virus HA peptide in the study.

Can f 1 peptide-specific TCLs were obtained about twice more frequently than TL peptide-specific TCLs from allergic subjects (1.85±0.32% vs. 0.74±0.17% positive TCLs out of 30 replicate wells per subject, Fig. 1A). The frequencies were comparable to those seen with the TCLs from nonallergic subjects (1.47±0.31% vs. 0.84±0.23% per subject, respectively). The numbers of the TCLs induced with Can f 1 and TL peptides did not correlate significantly in either of the subject groups (Spearman rank correlation). Importantly, the frequencies of lipocalin-specific TCLs clearly contrasted to those of HA peptide-specific TCLs (12.68±10.98% per allergic and 8.60±13.37% per nonallergic subject, respectively, Fig. 1A). The frequencies of TCLs specific to Can f 1 peptides, TL peptides and the HA peptide, respectively, did not differ between the two subject groups (Fig. 1A).

Based on the frequencies of specific TCLs obtained per subject (see above), we estimated the precursor frequencies of T cells specific to the Can f 1, TL and HA peptides in the circulating CD4^+^ T cell pool. When a total of three million PBMCs were seeded per peptide per person (30 wells, 10^5^ PBMCs per well), assuming that each peptide-specific TCL represents a monoclonal expansion of a specific CD4^+^ T cell and that PBMCs contain approximately 30% of CD4^+^ T cells, the average frequency of Can f 1 peptide-specific T cells was estimated to be 0.6 per 10^6^ CD4^+^ T cells in allergic subjects (0.0185×30 divided by 0.30×30×10^5^), ranging from 0 up to 1.6 per 10^6^ CD4^+^ T cells. In nonallergic subjects, the average frequency of Can f 1 peptide-specific T cells was estimated to be 0.5 per 10^6^ CD4^+^ T cells (from 0.2 up to 1.4 per 10^6^ CD4^+^ T cells). Similarly, the average frequencies for TL peptide-specific TCLs from allergic and nonallergic subjects, respectively, were 0.3 (0 to 0.6) and 0.3 (0 to 1.4) per 10^6^ CD4^+^ T cells. The figures for the HA peptide-specific TCLs were 4.2 and 2.9 per 10^6^ CD4^+^ T cells.

### The same individual Can f 1 and TL peptides are recognized by the TCLs of both allergic and nonallergic subjects

When we analyzed the epitope specificity of the TCLs generated with Can f 1 and TL peptide pools (Fig. 1B), specific T cell responses to almost all tested peptides were observed, with the exception of pC6, pTL8 and pTL9 within the allergic subjects and pTL5, pTL8 and pTL9 within the nonallergic subjects. In line with our previous study [30], pC4 was the most frequently recognized Can f 1 peptide by the TCLs of both allergic and nonallergic subjects (5.0±1.15% and 4.2±1.61%, respectively). Within the TL peptides, pTL3 was recognized the most frequently (1.89±0.57% and 4.2±0.68% in allergic and nonallergic subjects, respectively). Overall, no significant differences in the recognition of individual Can f 1 and TL peptides, respectively, were observed between the two subject groups.

When we directly compared the frequency of TCLs specific to each Can f 1 peptide to the respective frequency to its TL counterpart peptide, three peptides, pC4, pC7 and pC9, were observed to be recognized significantly more frequently than their counterpart TL peptides by the TCLs of allergic subjects (Fig. 1B). The phenomenon was not observed with the TCLs of nonallergic subjects (Fig. 1B).

In order to determine whether T cell cross-reactivity would account for the similarity of the responses to individual Can f 1 and TL peptides, the peptide-specific TCLs were stimulated with the counterpart Can f 1 or TL peptide up to a concentration of 10 µM. Only six Can f 1 peptide-specific TCLs (induced with pC1 or pC4) and one TL peptide-specific TCL (induced with pTL4) recognized the counterpart peptide in the proliferation assay. Five of the TCLs were obtained from allergic subjects and two from nonallergic subjects (Fig. S2).

### Strength of the CD4^+^ T cell response to Can f 1 and TL peptides is similar within the allergic or healthy subject group but differs between the groups

To characterize functionally the Can f 1, TL and HA-specific TCLs obtained, we analyzed their proliferative capacity *in vitro* upon stimulation with the peptides. Unexpectedly, the proliferative responses of allergic subjects' TCLs to TL peptides, in addition to those to Can f 1 peptides, were stronger than the responses of nonallergic subjects. This difference was the most evident at 0.1 µM (Fig. 2A) but also visible at higher peptide concentrations (Fig. S3). Interestingly, the proliferative responses did not differ between Can f 1 and TL peptide-specific TCLs within either of the subject groups. All these responses were, however, significantly weaker than those to the HA peptide in both subject groups (Fig. 2A).

The proliferative responses of allergic subjects' TCLs to one of the individual Can f 1 peptides, pC4, were found to be significantly stronger than those of healthy subjects' TCLs (Fig. S4). Of interest, the same phenomenon was observed with the homologous TL peptide, pTL4.

To analyze the functional avidity of the TCLs, an EC_50_ value for each peptide-specific TCL was determined, and the TCLs were categorized arbitrarily into groups of low (EC_50_>1.0 µM), intermediate (EC_50_ = 0.1–1.0 µM) and high avidity (EC_50_<0.1 µM). It turned out that only up to 1.4% of Can f 1 and up to 3.6% of TL peptide-specific TCLs were of high avidity (Fig. 2B), whereas a substantially greater proportion of HA peptide-specific TCLs from both allergic and control subjects were of high avidity (25.0% and 22.6%, respectively, Fig. 2B).

### Both Can f 1 and TL peptide-specific TCLs exhibit a Th2-biased phenotype whereas HA-specific TCLs exhibit a Th1-biased phenotype

To further functionally characterize the Can f 1, TL and HA-specific TCLs generated, the production of the cytokines IL-4, IL-5, IFN-γ and IL-10 was analyzed upon stimulation with 10 µM of the peptides. Both the Can f 1 and TL peptide-specific TCLs from allergic subjects produced more of the Th2-type cytokines IL-4 and IL-5 than the respective TCLs from nonallergic subjects (Fig. 3). When dissecting the cytokine production of TCLs specific to individual peptides, we found that the Th2-biased immune response of allergic subjects to Can f 1 mainly derived from a few individual epitopes, as the pC2, pC3 and pC4-specific TCLs of these subjects produced significantly more IL-4 and/or IL-5 than those of nonallergic ones (Fig. S5).

The Th1 bias of the cellular immune response of both allergic and healthy subjects to the HA peptide was obvious, as the HA peptide-specific TCLs produced significantly more IFN-γ than Can f 1 or TL peptide-specific TCLs (Fig. 3). Moreover, the Can f 1 and TL peptide-specific TCLs of allergic subjects produced more IL-4 and IL-5 than their HA peptide-specific TCLs.

To some extent, the production of IL-10 appeared to parallel that of the Th2-type cytokines, in particular when the TCLs of allergic and nonallergic subject groups with TL specificity were compared (Fig. 3). While the production of IL-4, IL-5 and IFN-γ by the Can f 1 and TL peptide-specific TCLs did not statistically differ within either of the subject groups, a higher level of IL-10 was produced by nonallergic subjects' Can f 1 peptide-specific TCLs than their TL peptide-specific TCLs.

Finally, we assessed the phenotypes of the generated TCLs by their surface marker expression. In accordance with the cytokine data, we found that the Th2-associated chemokine receptor CCR4 [38], [39] was expressed more and the Th1-associated chemokine receptor CXCR3 less on the Can f 1 and TL peptide-specific TCLs than on the HA peptide-specific TCLs of both allergic and nonallergic subjects (Figs. 4 and S6).

### Binding of the Can f 1, TL and HA peptides to HLA class II molecules

Differences in the binding affinities of the Can f 1, TL and HA peptides to HLA class II alleles could potentially account for the stimulatory capacities of the peptides. To address this possibility, we measured the binding of the peptides to 10 prevalent HLA class II molecules. The results are expressed as relative binding ratios in Figure 5.

We observed that every HLA molecule tested was capable of binding at least one Can f 1 and TL peptide with high affinity. When the binding capacity of each HLA molecule was compared between Can f 1 and TL peptides, none of them bound Can f 1 or TL peptides preferentially. When the binding affinity of all HLA alleles was compared between each pair of the peptides, for example, between pC1 and pTL1, a total of 9 pairs (Fig. 5), it was found that only pC7 of the Can f 1 peptides exhibited significantly higher affinity for HLA alleles than the counterpart peptide pTL7. Interestingly, the binding affinity of one of the TL peptides, pTL3, was also significantly higher than that of the counterpart peptide, pC3. The HA control peptide was observed to be bound with high affinity to five HLA molecules, which is a figure not atypical with some of the Can f 1 or TL peptides (Fig. 5). When the binding affinity of all HLA alleles was compared between the HA peptide and individual lipocalin peptides, the HA peptide bound to HLA significantly more strongly than one of the Can f 1 peptides, pC9, and three of the TL peptides, pTL2, pTL8 and pTL9.

## Discussion

The allergenicity of proteins is still an unresolved issue. We have previously proposed that the allergenicity of lipocalin allergens may be associated with the presence of homologous endogenous lipocalin proteins in humans resulting in the absence of high-avidity lipocalin allergen-reactive CD4^+^ T cells due to thymic deletion [6], [40]–[42]. Consequently, exogenous lipocalin allergens would be recognized suboptimally by CD4^+^ T cells, a phenomenon known to favor Th2 immune deviation [43]–[53]. In contrast with this view, it was recently proposed that immunogenicity would be a major factor accounting for the allergenicity of proteins [19]. In an attempt to clarify this discrepancy, we have focused here on comparing *in vitro* CD4^+^ T cell responses to dog Can f 1, a major lipocalin allergen, with those to a homologous human protein, tear lipocalin (TL).

Based on the estimation by the split-well method (Fig. 1A and B), we found that the frequency of Can f 1-specific CD4^+^ T cells in allergic and healthy subjects is very low, at the level of 1 in 10^6^ CD4^+^ T cells. This result is in line with our previous estimations on lipocalin allergen-specific CD4^+^ T cells, analyzed with the same method [34], [54], [55] or with peptide-HLA class II tetramers [55]. Exploiting the latter methodology, other investigators have reported similar or higher frequencies for CD4^+^ T cells specific to the cat allergen Fel d 1 [56], the birch pollen allergen Bet v 1 [57], [58] or the mite allergens Der p 1 and 2 [59].

An interesting finding in this study is that the frequency of Can f 1-peptide specific CD4^+^ T cells in both subject groups is close to the frequency of T cells specific to TL, an endogenous human homologue (Fig. 1A). These frequencies clearly contrast that of T cells specific to the single influenza virus HA peptide, known to contain a potent epitope [32]. The slightly higher frequency of Can f 1 peptide-specific TCLs over TL peptide-specific TCLs suggests, especially in allergic subjects, that Can f 1 peptide-specific T cells have expanded *in vivo* upon allergen exposure (Fig. 1A). Especially in allergic subjects, the phenomenon could be tracked to a few peptides, pC4, pC7 and pC9, which may indicate that these peptides play a more significant role in the allergenic T cell response (Fig. 1B). As HLA binding was found to be very similar between Can f 1 and TL peptides, and the distribution of HLA class II alleles did not differ between allergic and healthy subjects, it is an unlikely factor accounting for the results. It is conceivable, however, that the good stimulatory potential of pTL3 is associated with its good HLA-binding capacity (Fig. 5).

In accordance with the frequency data, we observed that the strength of *in vitro* proliferative responses of peptide-specific TCLs did not differ between Can f 1 and TL but that they were clearly weaker than the responses of TCLs specific to the HA peptide (Fig. 2A). A further analysis suggested that the weaker proliferative responses of Can f 1 and TL peptide-specific TCLs are attributed to the low frequency of TCLs with high functional TCR avidity (Fig. 2B). These results are in line with reports from other investigators, as they indicate that T cells specific to self antigens exhibit low TCR avidity whereas those specific to microbial antigens exhibit higher TCR avidity [60]–[62].

A surprising observation was that the proliferative responses of not only Can f 1 but also TL peptide -specific TCLs from allergic subjects were stronger than those of the TCLs from healthy subjects (Fig. 2A). Whereas *in vivo* selection and expansion [63] may explain the stronger Can f 1 responses of allergic subjects over those of healthy subjects, this explanation is harder to adapt to the stronger responses of allergic subjects to TL, an endogenous protein. One possibility is that there does exist a connection between the T cell reactivity to the epitope-containing Can f 1 peptides and the homologous TL peptides. For example, we found here that TL peptides bound to the 10 prevalent HLA class II molecules examined in a comparable manner as Can f 1 peptides (Fig. 5). It can also be speculated that the TL-specific T cells we obtained were still primed *in vivo* with Can f 1 (or other lipocalin allergen) peptides, even though we could demonstrate direct T cell cross-reactivity only in few cases between the peptides in our experimental setting. Of interest, we showed in our previous study with two Can f 1 peptides that one of them, which corresponds to pC7 in this study, induced from a dog-allergic patient two TCLs that cross-reacted with the counterpart TL peptide [54]. Hence, T cell cross-reactivity between Can f 1 and TL peptides obviously exists in humans. Finally, it is of interest that in our previous study, the sera of about half of the dog-allergic subjects with Can f 1-specific IgE were found to have TL-specific IgE, whereas no allergic subject with specific IgE to other lipocalin allergens had the latter antibodies [27]. Further studies showed that the specific IgE antibodies were cross-reactive to TL and Can f 1, respectively [27]. However, it is important to note that the IgE reactivity to TL, as assessed by IgE ELISA or skin prick tests in a limited number of Can f 1-allergic subjects (Table S1 and [27]), is very weak compared to the reactivity against Can f 1. This suggests that regardless of the shown similarities between the T cell responses to Can f 1 and TL peptides, strong IgE responses basically arise to the exogenous protein only. The marginally stronger antigenicity of Can f 1 peptides over those of TL peptides can be a factor contributing to this development.

Analysis of the phenotype of the Can f 1, TL and HA peptide-specific TCLs by cytokine production (Fig. 3) and chemokine receptor expression (Fig. 4) revealed both similarities with the proliferative responses of the TCLs (Fig. 2) and some unique features associated with atopic status and/or specificity for peptides. As with the proliferative responses, the production of IL-4 and IL-5 by both Can f 1 and TL-specific TCLs from allergic subjects was greater than that by the TCLs from nonallergic subjects (Fig. 3). The difference focused on a few peptides, including pC4 (Fig. S5), which again may be an indication of its significance in the allergenic immune response to Can f 1. Largely, these results appeared to reflect the atopic status of the subjects, and its influence was not observable with the HA-specific TCLs (Fig. 3). Again, as with the proliferative responses, the production of the Th2 cytokines did not significantly differ between Can f 1 and TL-specific TCLs within subject groups. Especially with the TCLs of nonallergic subjects, it can be speculated that this alludes to the Th2-deviating potential of Can f 1 peptides. Interestingly, the higher expression of CCR4, a Th2-associated chemokine receptor [39] and lower expression of CXCR3, a Th1-associated chemokine receptor [38], by the Can f 1 and TL-specific TCLs of nonallergic and allergic subjects compared to their HA-specific TCLs further points to the Th2 bias favored by lipocalin peptides (Fig. 4). As to IL-10, its production appeared to parallel the production of IL-4 and IL-5 to some extent. Of note, IL-10 production was observed to be higher in Can f 1-specific TCLs than in TL-specific TCLs of nonallergic subjects. A comparable phenomenon was not observed with the other cytokines measured. Since higher IL-10 production has been linked with protective, nonallergenic T cell responses [64], [65], this finding may reflect the induction of Can f 1-specific tolerance in the nonallergic subjects. In all, the Th1–Th2 phenotype of the Can f 1 and HA peptide-specific T cells lines of allergic and healthy subjects largely reflects and is in accordance with the paradigm known to delineate responses to allergens and microbial antigens [66]. Importantly, the phenotype of Can f 1 and TL peptide-specific TCLs resembled each other and differed from that of HA peptide-specific TCLs.

As discussed above, the *in vitro* T cell responses against the peptides of Can f 1, a dog allergen, and TL, a homologous self antigen, resembled each other and were clearly weaker than those against the influenza virus HA peptide, evaluated by the capacity to induce specific TCLs or, in particular, to induce proliferation of the TCLs upon stimulation with the peptides. Importantly, this observation is well in line with previous findings on the antigenic/immunogenic properties of lipocalin allergens. For example, Bos d 2, a bovine lipocalin allergen, proved to be a weak immunogen in studies with several inbred mouse strains with different MHC backgrounds [67], [68]. In human studies, lipocalin allergen proteins, such as cow Bos d 2 [69], dog Can f 1 [30], [70], horse Equ c 1 [71] and rat Rat n 1 [72], have also been observed to be weakly stimulatory *in vitro* for the PBMCs of sensitized subjects. Of note, two T cell epitopes from two distinct lipocalin allergens, Bos d 2 and Can f 1, characterized in detail, were found to be suboptimal; optimal (or heteroclitic, that is, more potent than the natural one) peptide analogues (altered peptide ligands) of the natural epitopes stimulated human T cell clones at 10–100-fold lower concentrations than the natural ligands [73], [74]. The heteroclitic activity of these peptides was attributed to their stronger recognition by TCR; they bound to the restricting HLA class II molecules with affinities similar to those of the natural ligands. When the Bos d 2-specific T cell clones were stained with HLA class II tetramers the structural TCR avidity of the clones for the heteroclitic ligands was found to be higher than that for the natural one [75], confirming the suboptimal characteristic of the natural epitope.

Considering all this information, strong antigenic/immunogenic capacity most probably is not the basis for the allergenicity of lipocalin allergens. In accordance with this conception, there are several reports indicating that *in vitro* T cell responses to an allergen do not correlate (or correlate very weakly) with the production of specific IgE in humans [23]–[25]. Therefore, poor T cell recognition, together with inefficient activation of the cells of innate immunity [76], also reported for several other allergens [77]–[79], appears as a likely immunological property of lipocalin allergens promoting their allergenicity since it is well documented that suboptimal T cell recognition of an antigen favors the development of Th2-type immune responses [43]–[53]. Nevertheless, good immunogenicity can be a characteristic for some other groups of allergens than lipocalins. For example, the supplemental information provided by da Costa Santiago et al. [19] shows that about 80% of the major allergens nonhomologous with human proteins, thereby probably immunogenic, were of plant origin. It is conceivable that these immunogenic plant proteins can act as allergens, as humans are probably exposed to these proteins together with Th2 immunity-promoting substances, known to be present in pollen [80]–[83]. In contrast, more than 80% of the major allergens homologous with human proteins were of animal or fungal origin, evolutionary closer to humans, and not apparently associated with adjuvant-like substances. In this group, the homology level of 30–40% with human proteins was considered optimal for the allergenicity of the proteins [19]. This is a finding of interest since lipocalin allergens in general exhibit homology with human proteins around this range [5], [6], [17].

Taken together, we have shown in this work that the major dog allergen Can f 1 and its human homologue TL resemble each other in several immunological properties. In particular, Can f 1 appears to be only marginally more potent to induce T cell responses than TL. As lipocalin allergens have also been shown to be weakly stimulatory to the cells of innate immunity [76], they may present themselves inert to the human immune system. When a protein with such characteristics encounters human atopic, Th2-biased immune environment, its subtle antigenicity may turn the fine balance of immunity for the favor of Th2 development. Therefore, we propose that specific CD4^+^ T cells should exhibit a certain avidity window for the epitopes of a homologous allergen to permit sensitization to take place: if the protein is recognized as truly foreign, a non-allergenic Th1-type response is likely; if it resembles the immunological self, the protein is probably tolerated; if it is able to cross the threshold for TCR activation, without being too stimulatory, a Th2-type immune response is possible [6].

## Supporting Information

Figure S1 **Peptides selected in the study.** Sequences of the Can f 1 peptides (pC1–C9) and the homologous tear lipocalin peptides (pTL1–pTL9) selected in the study are shown in grey boxes along the aligned Can f 1 and TL protein sequences. The selection was based on the seven Can f 1 sequences (black lines) previously verified to contain T cell epitopes of Can f 1 [30]. Additions of 1–3 amino acids to the ends of the peptides were made to include overlapping predicted HLA-DR-binding sites (sequences in blue, the P1 binding pockets in red). The panel was complemented with two peptides pC5/pTL5 and pC6/pTL6 predicted to contain HLA-DR-binding epitopes of both Can f 1 and TL.
(PDF)

Figure S2 **Cross-reactive T cell lines.** The proliferative responses of five cross-reactive TCLs from two allergic subjects (A-3, A-5) and those of two cross-reactive TCLs from two nonallergic subjects (NA-4, NA-6). The responses upon stimulation with the peptides used in the induction of the TCLs (▪) and with the cross-reactive counterpart peptides (□) at 10 µM are expressed as ΔCPM.
(TIF)

Figure S3 **Proliferation of TCLs specific to Can f 1, TL and HA peptides at 1 µM and 10 µM of antigen.** Proliferative responses of TCLs specific to 9 Can f 1 and 9 TL peptides and the HA peptide from allergic subjects (•) and nonallergic subjects (○) upon stimulation with the peptides at 1 µM (**A**) and 10 µM (**B**). The responses are expressed as ΔCPM (mean CPM of wells stimulated with the peptide - mean CPM of unstimulated wells).
(TIF)

Figure S4 **Proliferation of TCLs specific to pC4 and pTL4.** The proliferative responses of TCLs specific to the Can f 1 peptide pC4 and the counterpart tear lipocalin peptide pTL4 of allergic (•) and nonallergic subjects (○) are expressed as ΔCPM.
(TIF)

Figure S5 **Cytokine production by TCLs specific to the peptides pC2, pC3 and pC4.** Production of IL-4 and IL-5 by the TCLs specific to pC2, pC3 and pC4 from allergic (•) and nonallergic (○) subjects.
(TIF)

Figure S6 **Histogram presentation of the CCR4 and CXCR3 chemokine receptor expression by peptide-specific TCLs.** The expression of CCR4 and CXCR3 by a Can f 1 (pC9) and a HA peptide-specific TCL from the allergic subject A-12 is shown as a representative example of the effect of lipocalin vs. microbial peptide stimulation on the surface marker phenotypes of the TCLs.
(TIF)

Table S1 **Subjects enrolled in the study.**
(XLSX)
