# Supplementary figures and images for: Human CD4+ T Cell Responses to the Dog Major Allergen Can f 1 and Its Human Homologue Tear Lipocalin Resemble Each Other
Source: PLoS One. 2014 May 29;9(5):e98461. doi: 10.1371/journal.pone.0098461 (PMC4038554; doi:10.1371/journal.pone.0098461)

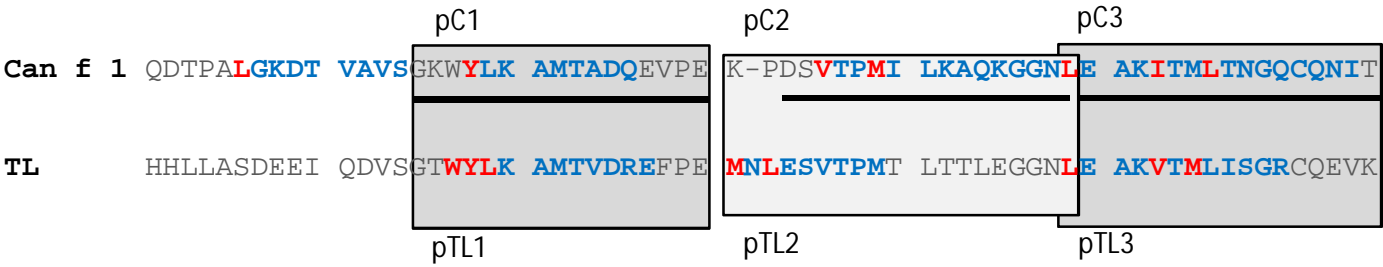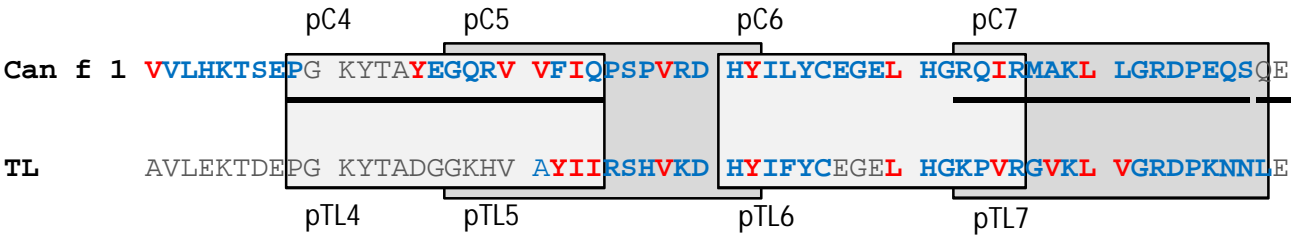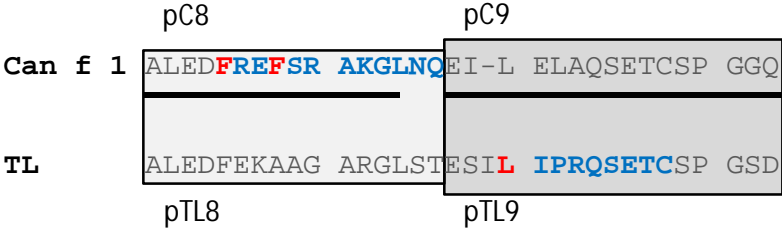

Supplement: Figure S1 — Peptides selected in the study. Sequences of the Can f 1 peptides (pC1–C9) and the homologous tear lipocalin peptides (pTL1–pTL9) selected in the study are shown in grey boxes along the aligned Can f 1 and TL protein sequences. The selection was based on the seven Can f 1 sequences (black lines) previously verified to contain T cell epitopes of Can f 1 [30]. Additions of 1–3 amino acids to the ends of the peptides were made to include overlapping predicted HLA-DR-binding sites (sequences in blue, the P1 binding pockets in red). The panel was complemented with two peptides pC5/pTL5 and pC6/pTL6 predicted to contain HLA-DR-binding epitopes of both Can f 1 and TL. (PDF) [file pone.0098461.s001.pdf]

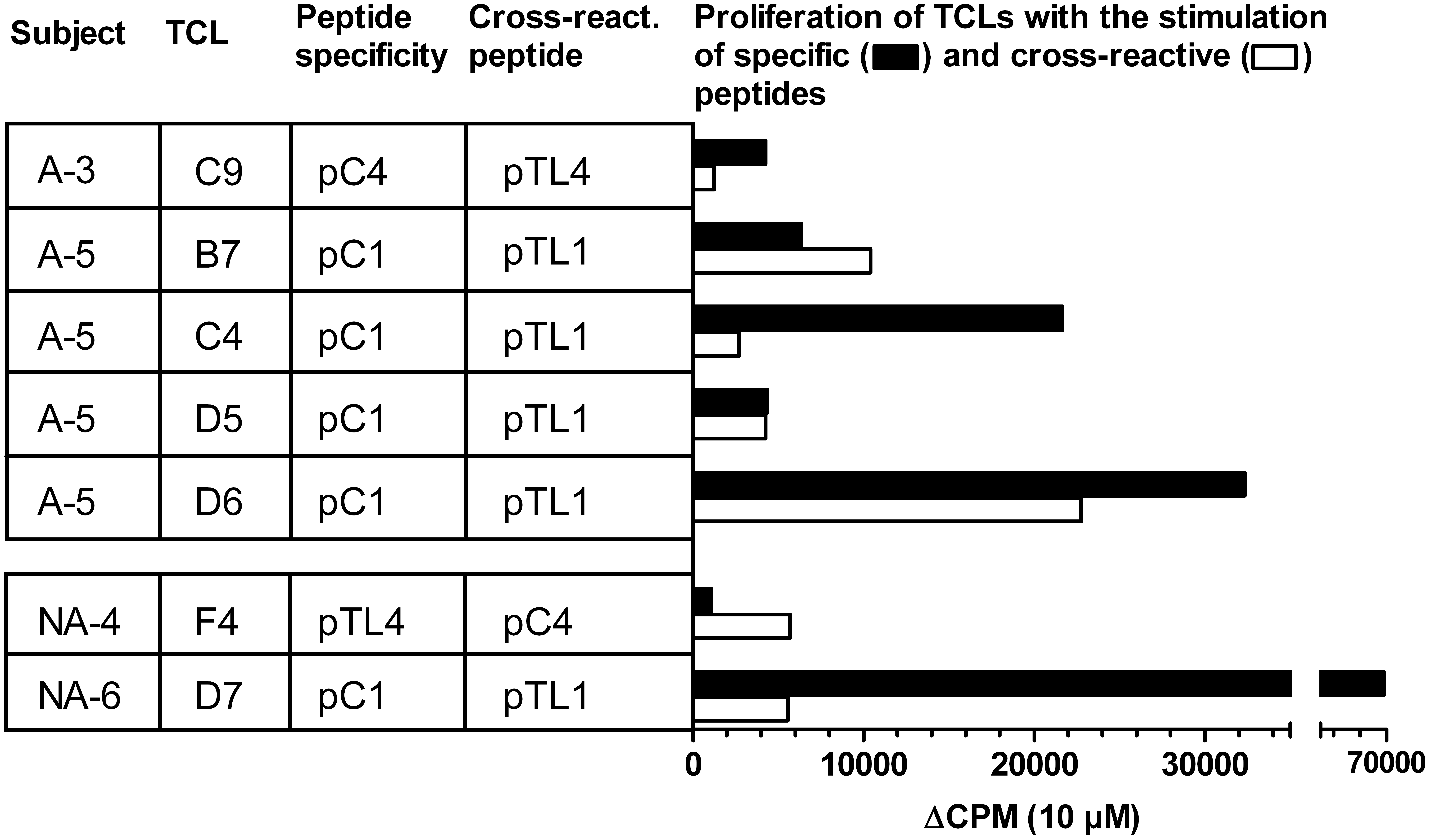

Supplement: Figure S2 — Cross-reactive T cell lines. The proliferative responses of five cross-reactive TCLs from two allergic subjects (A-3, A-5) and those of two cross-reactive TCLs from two nonallergic subjects (NA-4, NA-6). The responses upon stimulation with the peptides used in the induction of the TCLs (▪) and with the cross-reactive counterpart peptides (□) at 10 µM are expressed as ΔCPM. (TIF) [file pone.0098461.s002.tif]

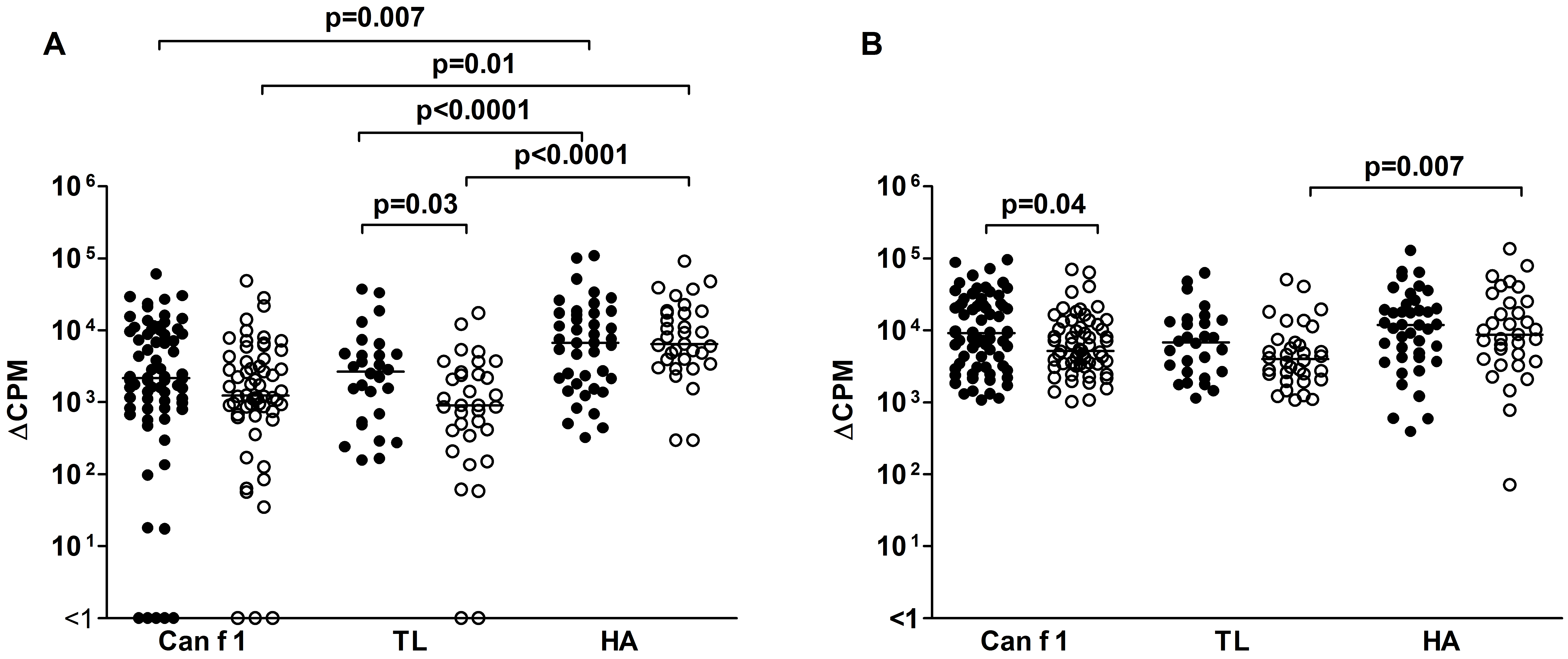

Supplement: Figure S3 — Proliferation of TCLs specific to Can f 1, TL and HA peptides at 1 µM and 10 µM of antigen. Proliferative responses of TCLs specific to 9 Can f 1 and 9 TL peptides and the HA peptide from allergic subjects (•) and nonallergic subjects (○) upon stimulation with the peptides at 1 µM (A) and 10 µM (B). The responses are expressed as ΔCPM (mean CPM of wells stimulated with the peptide - mean CPM of unstimulated wells). (TIF) [file pone.0098461.s003.tif]

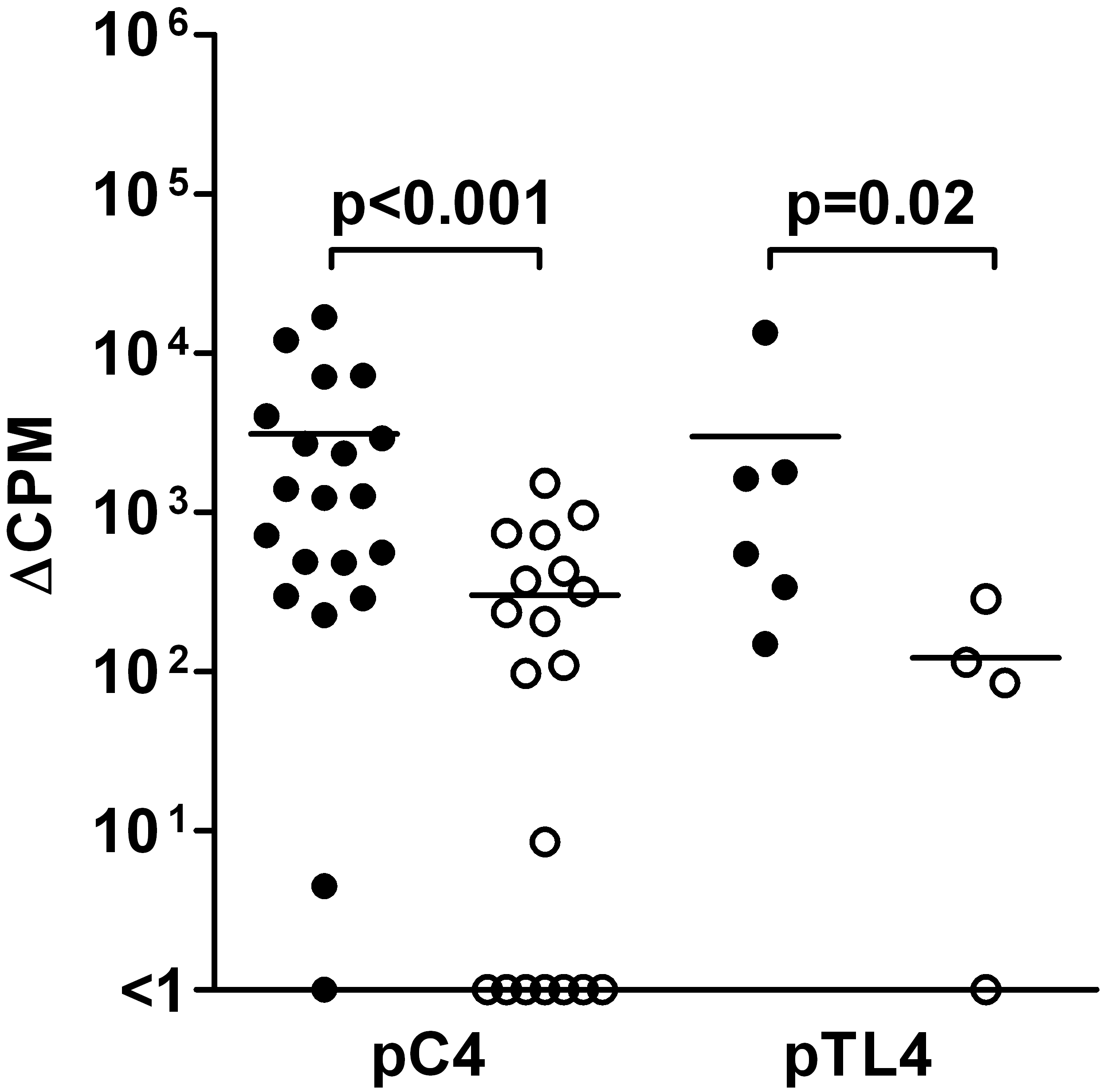

Supplement: Figure S4 — Proliferation of TCLs specific to pC4 and pTL4. The proliferative responses of TCLs specific to the Can f 1 peptide pC4 and the counterpart tear lipocalin peptide pTL4 of allergic (•) and nonallergic subjects (○) are expressed as ΔCPM. (TIF) [file pone.0098461.s004.tif]

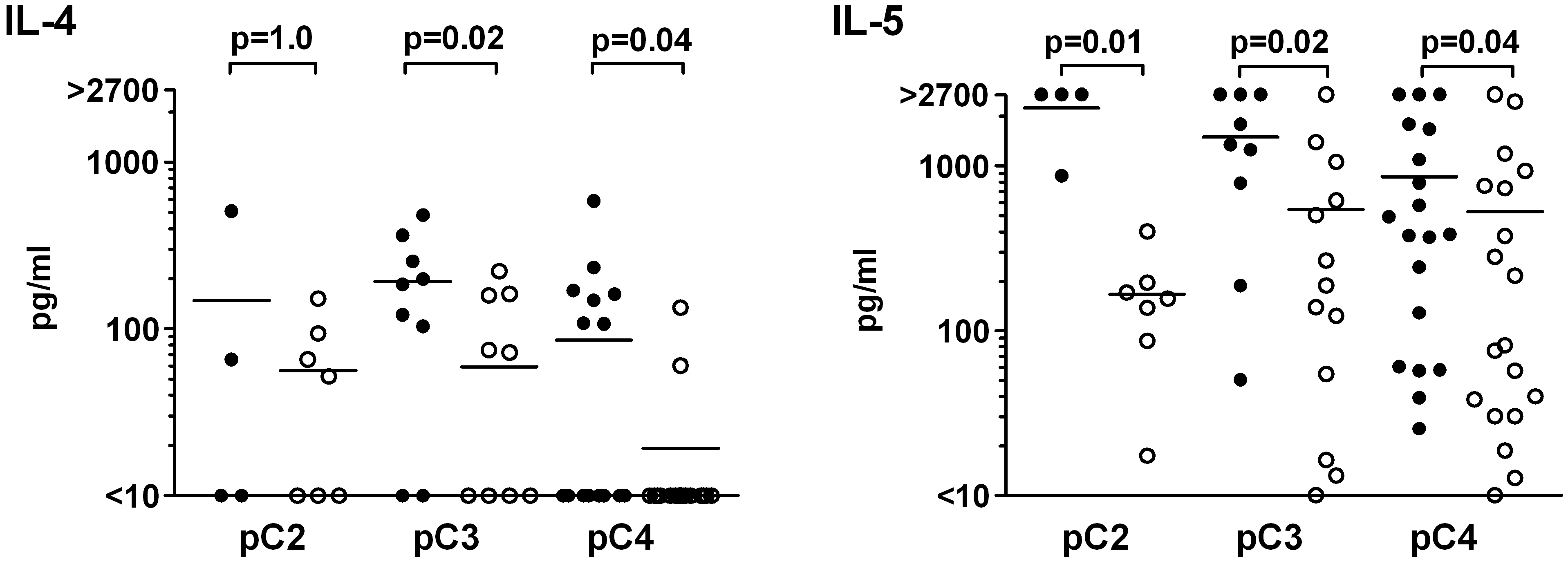

Supplement: Figure S5 — Cytokine production by TCLs specific to the peptides pC2, pC3 and pC4. Production of IL-4 and IL-5 by the TCLs specific to pC2, pC3 and pC4 from allergic (•) and nonallergic (○) subjects. (TIF) [file pone.0098461.s005.tif]

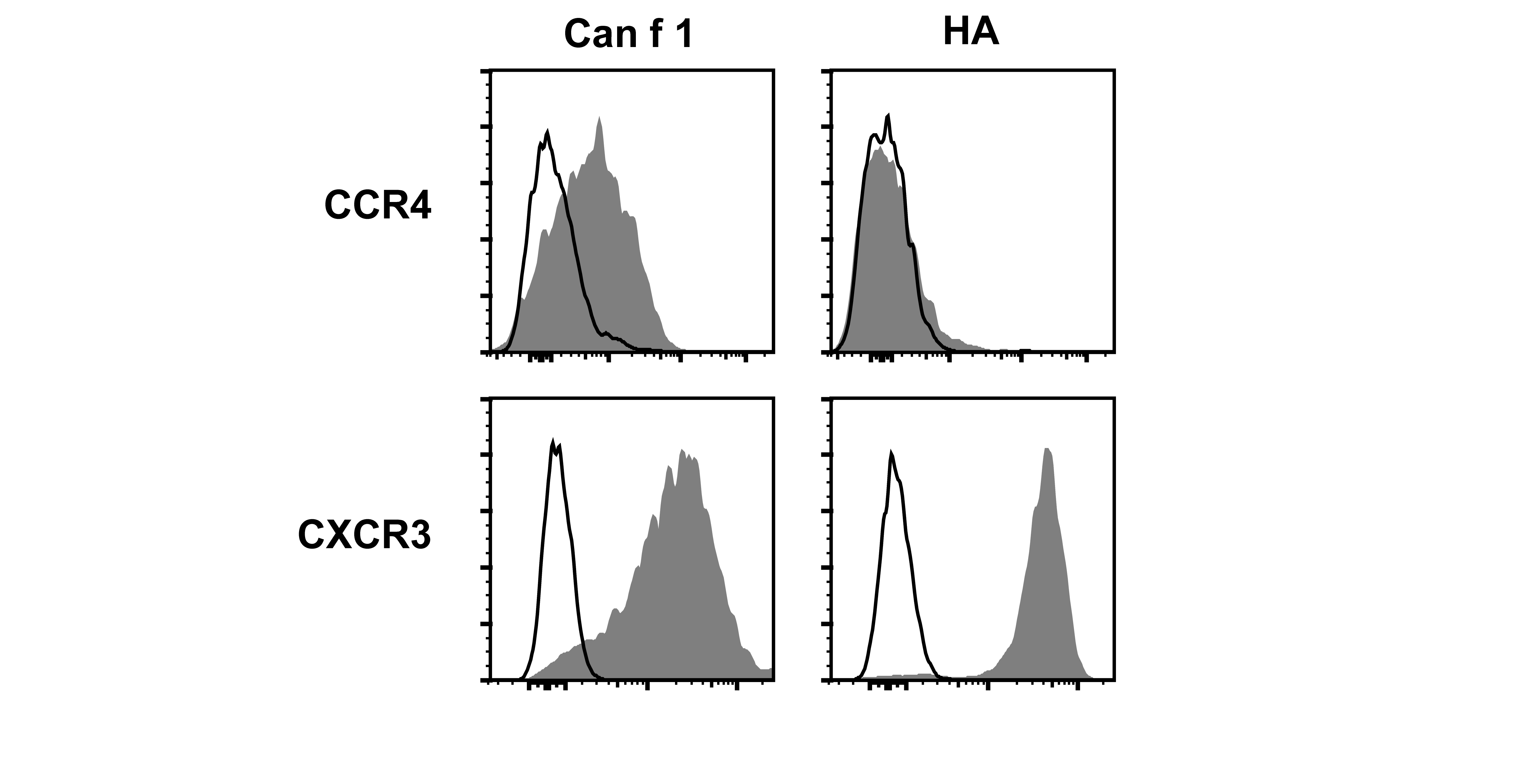

Supplement: Figure S6 — Histogram presentation of the CCR4 and CXCR3 chemokine receptor expression by peptide-specific TCLs. The expression of CCR4 and CXCR3 by a Can f 1 (pC9) and a HA peptide-specific TCL from the allergic subject A-12 is shown as a representative example of the effect of lipocalin vs. microbial peptide stimulation on the surface marker phenotypes of the TCLs. (TIF) [file pone.0098461.s006.tif]
